# The Mechanism of Aniline Blue Degradation by Short-Chain Dehydrogenase (*SDRz*) in *Comamonas testosteroni*

**DOI:** 10.3390/molecules29225405

**Published:** 2024-11-15

**Authors:** Chuanzhi Zhang, Yong Huang, Jiaxin He, Lei He, Jinyuan Zhang, Lijing Yu, Elshan Musazade, Edmund Maser, Guangming Xiong, Miao Xu, Liquan Guo

**Affiliations:** 1 College of Life Sciences, Jilin Agricultural University, Changchun 130118, China; zcz100@126.com (C.Z.); armyfist@163.com (Y.H.); hjx13104411648@163.com (J.H.); 15164304170@163.com (L.H.); zhangjinyuan6959@163.com (J.Z.); elshan.musazade1@gmail.com (E.M.); 2 School of Grain Science and Technology, Jilin Business and Technology College, Changchun 130507, China; 3 School of Food and Biology, Changchun Polytechnic, Changchun 130033, China; xiayu8054@163.com; 4 Institute of Toxicology and Pharmacology, University Medical School Schleswig-Holstein, 24105 Kiel, Germany; maser@toxi.uni-kiel.de (E.M.); xiong-guangming@outlook.com (G.X.)

**Keywords:** *Comamonas testosteroni*, short-chain dehydrogenase, aniline blue, biodegradation

## Abstract

Dye wastewater pollution, particularly from persistent and toxic polycyclic organic pollutants, such as aniline blue, poses a significant environmental challenge. Aniline blue, a triphenylmethane dye widely used in the textile, leather, paper, and pharmaceutical industries, is notoriously difficult to treat owing to its complex structure and potential for bioaccumulation. In this study, we explored the capacity of *Comamonas testosteroni* (CT1) to efficiently degrade aniline blue, focusing on the underlying enzymatic mechanisms and degradation pathways. Through prokaryotic transcriptome analysis, we identified a significantly upregulated short-chain dehydrogenase (*SDRz*) gene (log_2_FC = 2.11, *p* < 0.05) that plays a crucial role in the degradation process. The SDRz enzyme possessed highly conserved motifs and a typical short-chain dehydrogenase structure. Functional validation using an *SDRz*-knockout strain (CT-ΔSDRz) and an *SDRz*-expressioning strains (E-SDRz) confirmed that SDRz is essential for aniline blue degradation. The knockout strain CT-ΔSDRz exhibited a 1.27-fold reduction in the degradation efficiency, compared to CT1 strain after 12 h; while the expression strain E-SDRz showed a 1.24-fold increase compared to *Escherichia coli* DH5α after 12 h. Recombinant SDRz (rSDRz) was successfully produced, showing significant enzymatic activity (1.267 ± 0.04 mmol·L^−1^·min^−1^ protein), with kinetic parameters Vmax = 2.870 ± 0.0156 mmol·L⁻^1^·min⁻^1^ protein and Km = 1.805 ± 0.0128 mM·mL^−1^. Under optimal conditions, the rSDRz achieved a degradation efficiency of 62.17% for aniline blue. Gas chromatography–mass spectrometry (GC-MS) analysis identified several intermediate metabolites in the degradation pathway, including benzeneacetaldehyde, a, a-diphenyl, 2-amino-4-methylbenzophenone, benzene, 1-dimethylamino-4-phenylmethyl, benzenesulfonic acid, methyl ester, further elucidating the biodegradation mechanism. These findings highlight SDRz as a critical enzyme in the biodegradation of aniline blue, offering valuable insights and a robust theoretical foundation for developing advanced bioremediation strategies to address dye wastewater pollution.

## 1. Introduction

Dye wastewater pollution is one of the most significant types of water pollution, and dye wastewater treatment is a challenging problem in the sewage treatment industry [1]. Aniline blue, a triphenylmethane dye, is a large organic molecule with a multi-benzene ring structure, widely used in the textile, leather, paper, and pharmaceutical industries [2,3]. Polycyclic organic pollutants in water are difficult to degrade, have bioaccumulation potential, and exhibit “three-causes” toxicity (carcinogenic, mutagenic, and teratogenic effects), making them the primary pollutants in water [4]. Therefore, the degradation and removal of triphenylmethane dye pollutants from water bodies has become a frontier topic in biological and environmental sciences.

Adsorption and photocatalysis are effective techniques for removing aniline blue pollution from printing and dyeing wastewater [5,6]. Biological treatment methods are considered one of the best choices for treating organic pollutants in environmental water because of their low cost, thorough mineralization, strong resistance to water quality fluctuations, and environmental friendliness [7,8]. There are two main mechanisms of microbial degradation of aniline blue: (1) Extracellular enzymes secreted by microorganisms degrade aniline blue. (2) Aniline blue is reduced by electron transfer through the Mtr respiratory pathway of microorganisms [9]. Bacteria known to decolorize and degrade aniline blue include *Shewanella oneidensis* MR-1, *Streptomyces* AG-56, *P. aeruginosa* WZR-B, *Providencia* sp. EL2, *Acinetobacter* sp. EL1, *Weeksella virosa* EL3 and *Lysinibacillus fusiformis* N019a. This process is primarily driven by enzymes such as azoreductase, triarylmethane reductase (TMR), dioxygenase, and the decolorizing enzyme TpmD [9,10,11,12,13,14]. Currently, most studies on the bacterial degradation of dye pollutants have focused on the biodegradation of azo and anthraquinone dyes. However, as a triphenylmethane dye, aniline blue is challenging to biodegrade because of its high molecular weight, complex structure, and numerous unsaturated bonds. This leads to slow research progress and a lack of clarity in degradation pathways and molecular mechanisms.

*Comamonas testosteroni* is an aerobic, motile, nonspore-forming, ubiquitous Gram-negative organism, and is distributed widely in soil, sediments, and garden ponds [15,16]. It has low virulence potency and has been rarely observed as an infectious agent in clinical practice. There are few reports on its aggressive manner as an opportunistic pathogen [15,17]. *Comamonas testosteroni* strains could efficiently degrade polycyclic aromatic hydrocarbons (PAHs) in contaminated soil [18], they can be considered a promising candidate for bioremediation processes due to their ability to degrade various organic pollutants [19,20]. Previous studies have shown that among many polycyclic compound degradation processes, *Comamonas testosteroni* CT1 (ATCC11996) exhibits highly efficient degradation of polycyclic aromatic hydrocarbons (PAHs) [21,22,23]. Short-chain dehydrogenase/reductase (SDR) is considered a key enzyme initiating the bacterial degradation of PAHs [24]. In the metabolic processes of many large organic compounds, reducing carbonyl compounds, and including aldehydes, ketones, and quinones to their corresponding hydroxyl derivatives is crucial [25]. Hydroxy and carbonyl groups are the most common chemical groups SDR substrates target for conversion. SDR enzymes also catalyze the reduction in C=C and C=N (chromophore group) double bonds, mediating dehydratase, sulfotransferase, isomerase, and decarboxylation reactions [26]. Studies have identified that its substrates encompass aliphatic aldehydes and ketones, monosaccharides, steroids, prostaglandins, flavonoids, PAHs, retinoids, and numerous other important biological products [25,27,28,29]. Research indicates that the *SDR* gene functions as an oxidoreductase, utilizing NAD^+^ or NADP^+^ as cofactors to act on donor CH-OH groups [30,31]. The SDR enzyme plays a crucial role in metabolic processes and holds significant research value.

Aniline blue has a stable aromatic ring structure. The natural strains with high efficiency degradation of aromatic cyclic substances were selected as the experimental materials. This study identified seven natural bacterial strains capable of efficiently degrading PAHs: *C. testosteroni* CT1 and KF-1, *Buttiauxella* sp. S19-1, *P. putida* PS, *P. stutzeri* JP1, *Pseudomonas* sp. LY1, and *Acinetobacter calcoaceticus* LM1. Among these, CT1 was the most effective in degrading aniline blue. Through prokaryotic transcriptomic analysis, we found a new *SDR* gene (named *SDRz*) with significant expression difference in the degradation of aniline blue, indicating that *SDRz* gene is a key gene in the degradation of aniline blue by CT1 bacteria. Therefore, this study conducted molecular biology research and functional verification of *SDRz*, studying its catalytic function and enzymatic properties and examining the degradation products of aniline blue by recombinant SDRz (rSDRz). This study aimed to elucidate the role of SDRz in the metabolic degradation of aniline blue.

## 2. Results and Discussion

### 2.1. Biodegradation Ability of Different Strains to Aniline Blue

**Figure 1:**
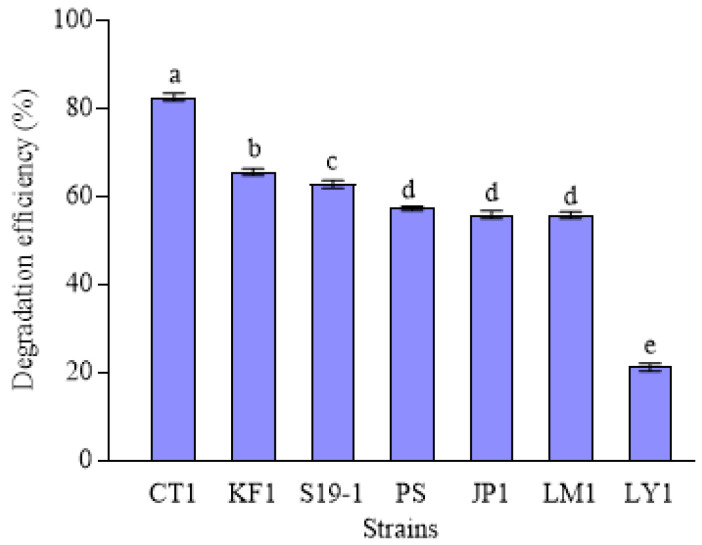
Seven macrocyclic-degrading bacterial strains were capable of degrading aniline blue. Each data point represents N = 3, with values expressed as mean ± standard deviation ($\bar{x}$ ± SD). Statistical significance was determined at *p* < 0.05.

The biodegradation of 200 mg·L^−1^ aniline blue by the seven natural strains (CT1, KF1, S19-1, PS, JP1, LM1, and LY1) was shown in Figure 1. Among these, CT1 exhibited the highest degradation efficiency (82.56%). KF1 exhibited a degradation efficiency of 65.51%. The 16S rRNA gene sequence of strain KF1 is 100% homologous to *C. testosteroni* CT1 (ATCC11996) [32]. KF1 can degrade sulfophenylcarboxylates (SPC), such as 3-(4-sulfophenyl)butyrate (3-C4-SPC) [33]. This strain encodes a large number of aromatic ring cleavage genes. However, it does not degrade substances such as benzenesulfonate, sulfoacetate, or oxalate, and lacks gene clusters for phenol and nitrobenzene degradation in its genome [34]. This could account for the decreased degradation efficiency of aniline blue in KF1 compared to strain CT1. Strains S19-1, PS, JP1, and LM1 showed similar degradation efficiency, ranging from 55.69% to 62.71%, whereas LY1 exhibited the lowest degradation efficiency of 21.15%. The lower degradation efficiency of these five strains indicated they were unsuitable for further studies on aniline blue degradation. Therefore, strain CT1 was selected for further experiments on the degradation of aniline blue.

### 2.2. Optimization of Conditions for Aniline Blue Biodegradation by Strain CT1

As shown in Figure S1A, strain CT1 demonstrated a solid ability to degrade aniline blue within a concentration range of 100 mg·L^−1^ to 2000 mg·L^−1^. At 32 °C, after 12 h of degradation, the degradation efficiency of 100 mg·L^−1^ and 200 mg·L^−1^ aniline blue were 92.45% and 92.25%, respectively. However, as the aniline blue concentration increased, the degradation efficiency decreased significantly. Therefore, a concentration of 200 mg·L^−1^ of aniline blue was chosen for further research. Temperature had a minimal effect on the degradation of aniline blue by strain CT1. As shown in Figure S1B, the degradation efficiency was highest at 92.64% at 32 °C, decreased to 87.39% at 37 °C, and was lowest at 82.02% at 22 °C. Therefore, the optimal degradation temperature was determined to be 32 °C. As shown in Figure S1C, the degradation efficiency of aniline blue increased over time, reaching 92.26% after 12 h. Subsequent increases in the degradation efficiency were minimal. Thus, 12 h was chosen as the optimal degradation time for this study.

Previous studies have shown that various microorganisms effectively degrade aniline blue. Wu et al. demonstrated that *S. oneidensis* MR-1 effectively degraded aniline blue at concentrations ranging from 20 mg·L^−1^ to 1000 mg·L^−1^. Their study showed that achieving over 90% degradation required between 36 and 96 h, highlighting the organism’s efficiency across a broad concentration range [35]. Another study found that *Mucoromycotina* sp. HS-3 can degrade 100 mg·L^−1^ aniline blue, achieving a decolorization rate of 95% after five days of static culture [36]. The degradation time for these microorganisms was much longer than that for strain CT1. Emtiazi et al. showed that *Cladosporium* sp. and *Fusarium* sp. could degrade aniline blue with 89% and 84% decolorization rates, respectively. These fungi can use aniline blue as the sole nitrogen source but not as the sole carbon source [37]. Ma et al. demonstrated that *Streptomyces* AG-56 achieved a decolorization rate of approximately 72% for aniline blue [38].

In this study, strain CT1 showed a wide range of adaptability to aniline blue concentrations, effectively degrading concentrations from 100 mg·L^−1^ to 2000 mg·L^−1^. The degradation time for aniline blue was shorter than that for fungi, achieving over 90% degradation within 12 h. The degradation efficiency surpassed that of most microorganisms, with an optimal temperature range from 27 to 32 °C, making it more suitable for application in environmental water bodies.

### 2.3. Screening of Key Genes Involved in Aniline Blue Degradation

**Figure 2:**
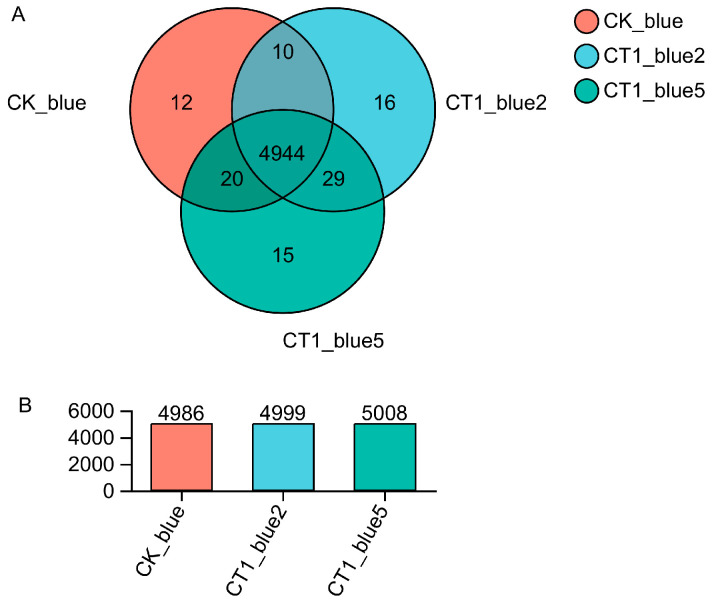
Analysis of gene expression of strain CT1. (**A**) Venn diagram of expressed genes shared among three groups which labeled on graph are defined as: CK_blue—control cultures, CT1_blue2—aniline blue concentrations of 200 m·L^−1^ treated cultures, CT1_blue5—aniline blue concentrations of 500 mg·L^−1^ treated cultures. All groups were cultured at 27 °C at 180 rpm for 12 h. (**B**) Gene expression profile.

**Figure 3:**
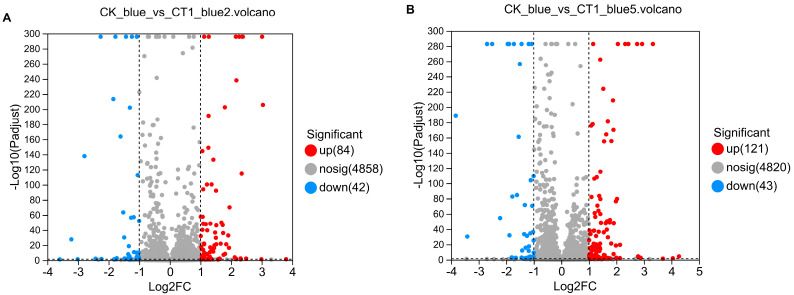
Volcano plots of differentially expressed genes. (**A**) Volcano plot between CK group and CT1_blue2 group; (**B**) Volcano plot between CK group and CT1_blue5 group.

Transcriptomic analysis of *C. testosteroni* CT1 during aniline blue degradation revealed 4944 DEGs across the three groups (Figure 2). Between CK_blue and CT1_blue2, 126 significant DEGs (log_2_FC > 1, *p* < 0.05) were identified, with 84 upregulated and 42 downregulated (Figure 3A). Similarly, 164 significant DEGs were observed between CK_blue and CT1_blue5, including 121 upregulated and 43 downregulated (Figure 3B).

**Figure 4:**
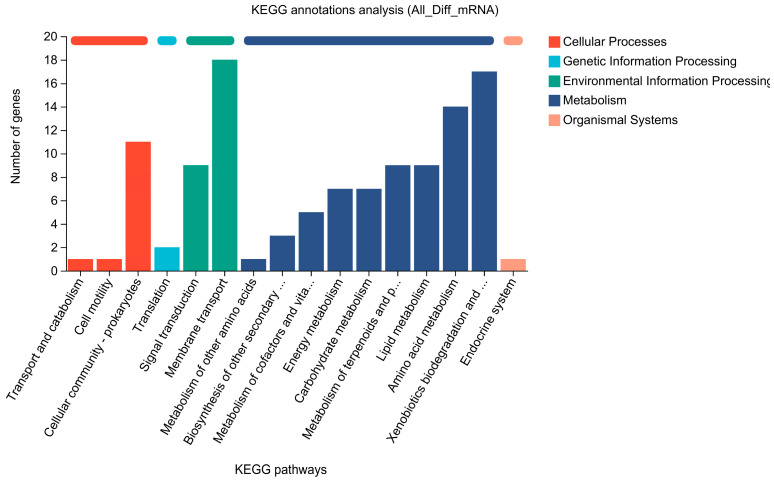
KEGG analysis of differentially expressed genes.

**Figure 5:**
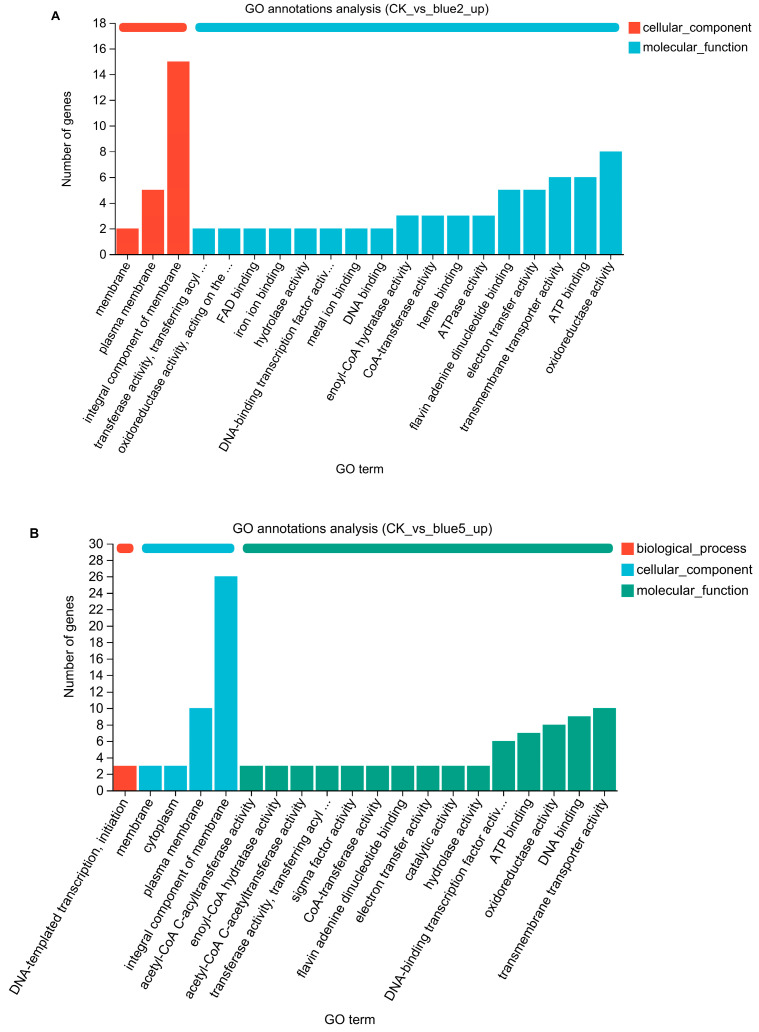
GO annotation analysis of up-regulated genes. (**A**) GO annotation analysis between CK group and CT1_blue2 group; (**B**) GO annotation analysis between CK group and CT1_blue5 group.

KEGG enrichment analysis indicated that 63 DEGs were enriched, with 17 DEGs involved in xenobiotic biodegradation and metabolism primarily related to aromatic compound metabolic pathways (Figure 4). GO annotation analysis showed that 96 significantly upregulated genes were shared between CK_blue and vs. CT1_blue2 (Figure 5A) and CK_blue vs. CT1_blue5 (Figure 5B) are mainly associated with molecular functions. Among these genes, eight are associated with oxidoreductase activity, including CTATCC11996_16599 (GenBank accession number: AHIL01000037, region: 89544...90314), which encodes an oxidoreductase from the SDR superfamily (WP_003078050.1). This gene, designated as *SDRz*, is 771 bp long and encodes a protein composed of 256 amino acids.

Transcriptomic analysis showed significant differences in *SDRz* gene expression between CT1 groups. Compared with the CK group, *SDRz* expression in the CT1_blue2 and CT1_blue5 groups increased by 2.11 and 1.89 times, respectively (Table S1). These results suggest that *SDRz* plays a crucial role in aniline blue biodegradation by the strain CT1.

### 2.4. Homology Analysis of SDRz

NCBI BLAST analysis revealed that *SDRz* shares 99% homology with the SDR gene from *C. testosteroni* G1 and over 92% homology with *SDR* genes from *C. testosteroni* YAZ2, T5-67, and MWF001. Multiple sequence alignment indicated that *SDRz* has secondary structures similar to *SDR* sequences in *Streptomyces*, *Soybean*, *Bacillales*, *Clostridioides difficile*, and *Pseudomonas* (Figure S2A). Sequence analysis (Figure S2B) showed that *SDRz* encodes two highly conserved SDR motifs: the G14xG16xG18 motif for coenzyme NAD(H) or NADP(H) binding and the NxSxVxK (112, 140, 153, 157) motif related to catalytic activity, classifying it as a short-chain alcohol dehydrogenase [26,39,40,41].

3D structural modeling using the SWISS-MODEL homology server, based on the *SDR* family oxidoreductase from *C. suwonensis* (A0A7X9U1F2_9BURK), indicated a confidence level of 0.96 for the SDRz model (Figure S3). Structural comparison with *C. testosteroni* TK8102 SDR (A0A076PML1) showed 100% similarity, exhibiting the classical three-dimensional structure of SDRs [26]. These findings indicate that SDRz shares similar sequences and structures with SDRs from different sources, suggesting that SDRs may have conserved catalytic activity.

### 2.5. Construction of SDRz-Expressing Strain

PCR amplified SDRz (sequence is shown in Figure S4A), and the product was verified using 1% agarose gel electrophoresis, as shown in Figure S4B. A pT-SDRz cloning vector was constructed (Figure S4C). Sequencing of the recombinant plasmid showed 100% similarity with the CTATCC11996_16599 gene (Figure S5), confirming the successful construction of the *SDRz* cloning vector. This vector was then transformed into *E. coli* DH5α cells, which were named E-SDRz.

### 2.6. Construction of SDRz Knockout Mutant and Gene Functional Verification

The CT1 strain was used as the template for PCR amplification of the Δ*SDRz* gene (Figure S6A). The presence of a single 401 bp band was confirmed by 1% agarose gel electrophoresis (Figure S6B). The Δ*SDRz* gene fragment was successfully amplified using PCR validation and sequencing alignment. The Δ*SDRz* gene was ligated into the pCR2.1-TOPO vector, sequenced, and aligned to construct the knockout vector pTOPO-ΔSDRz (Figure S6C). Moreover, the double transformation of pTOPO-ΔSDRz into strain CT1 was carried out via electrotransfection, following the methodology outlined by Xu et al. [22]. The pΔS-F′/pΔS-R′ primer pair was used to verify the genes in the recombinant strain. Agarose gel electrophoresis results and sequencing verification using CT-ΔSDRz (Figure S6D).

**Figure 6:**
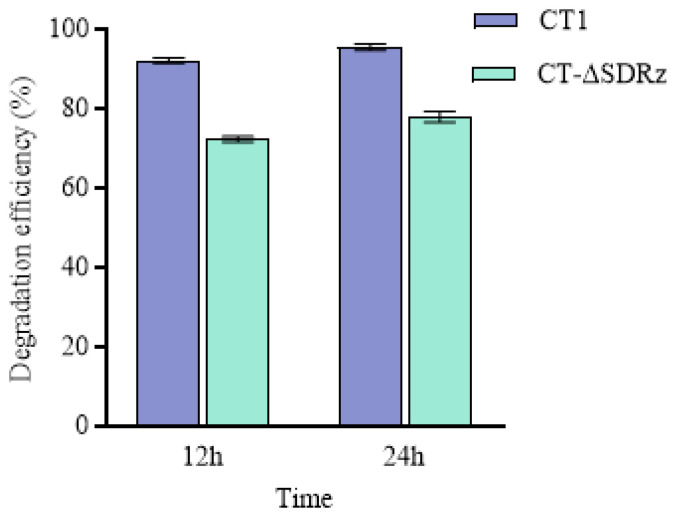
Aniline blue degradation by the wild-type CT1 and CT-ΔSDRz mutant.

CT-ΔSDRz and wild-type CT1 were tested separately for aniline blue degradation to verify gene function. As shown in Figure 6, with an aniline blue concentration of 200 mg·L^−1^ and a degradation time of 12 h at 32 °C, the degradation efficiency was 92.17% by strain CT1, while CT-ΔSDRz achieved 72.34%. After 24 h, the degradation efficiency of CT1 increased to 95.52%, whereas CT-ΔSDRz reached 77.96%. These results indicate that knocking out SDRz significantly reduces the ability of CT1 to degrade aniline blue.

**Figure 7:**
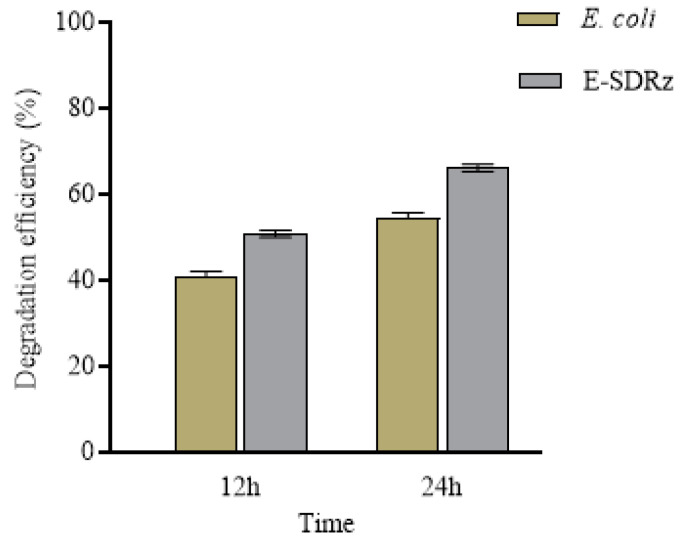
Aniline blue degradation by wild-type *E. coli* DH5α and the E-SDRz.

Comparing the differences in aniline blue degradation between *E. coli* DH5α and the E-SDRz after 12 h (Figure 7), *E. coli* achieved a degradation efficiency of 41.09%, whereas E-SDRz achieved 50.85%. After 24 h, the degradation efficiency of DH5α increased to 54.71%, whereas that of E-SDRz reached 66.17%. This suggests that the SDRz plays a critical role in the degradation of aniline blue.

### 2.7. Expression and Purification of rSDRz

The pT-SDRz vector was double-digested with *EcoR* I and *Hind* III (Figure S7A). The recombinant expression vector pET-SDRz was obtained following ligation and recombinant identification. The pET-SDRz vector was transformed into *E. coli* BL21 (DE3) cells to obtain an *SDRz* expression strain. Sequencing confirmed the successful construction of the recombinant expression vector (Figure S7B).

Under induction with 0.5 mM IPTG at 37 °C until the OD_600nm_ reached 0.6, followed by further induction at 20 °C for 20 h. The recombinant enzyme was purified using a Ni-TED agarose purification resin to obtain rSDRz. SDS-PAGE analysis revealed a single band for the purified protein (Figure S8). A standard curve was plotted using bovine serum albumin as a standard (Figure S9), determining the purified protein concentration to be 1.06 mg·mL^−1^.

In vitro enzymatic reaction results showed that using NADH as a coenzyme and aniline blue as a substrate, rSDRz exhibited enzymatic activity of 1.267 ± 0.04 mmol·L^−1^·min^−1^. Enzyme kinetic analysis of rSDRz is shown in Figure S10 with V_max_ = 2.870 ± 0.0156 mmol·L^−1^·min^−1^ protein and K_m_ = 1.805 ± 0.0128 mM·mL^−1^. These results demonstrate the successful construction of an SDRz recombinant expression vector and the functional role of SDRz in aniline blue degradation.

### 2.8. Analysis of Aniline Blue Degradation Products by rSDRz

UV-visible spectrophotometry was used to measure the effect of aniline blue degradation by rSDRz. At a concentration of 200 mg·L^−1^ aniline blue at 27 °C and rSDRz incubation for 5 min, the degradation efficiency of aniline blue reached 62.17%. This indicates that the SDRz is involved in the initial stages of aniline blue degradation by CT1.

**Figure 8:**
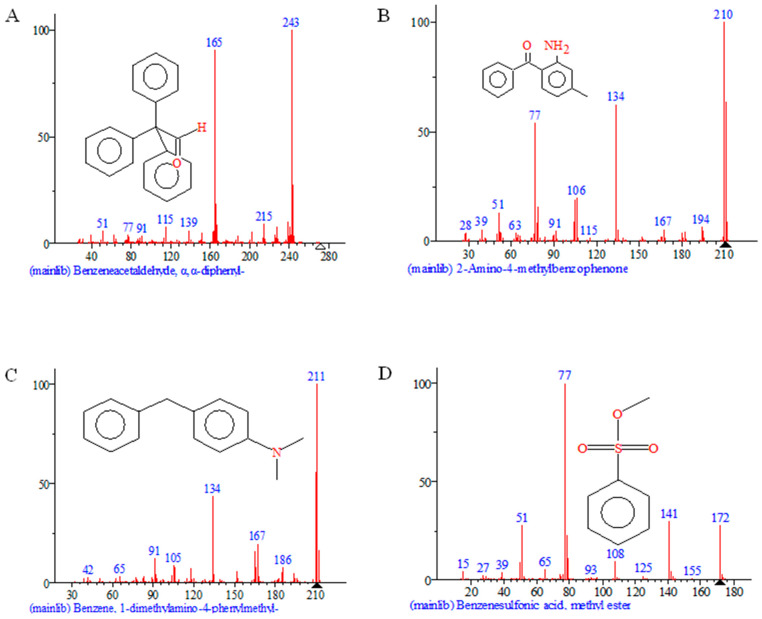
GC-MS analysis of aniline blue metabolites after degradation by rSDRz for 5 min. (**A**) benzeneacetaldehyde, a, a-diphenyl; (**B**) 2-amino-4-methylbenzophenone; (**C**) benzene, 1-dimethylamino-4-phenylmethyl; (**D**) benzenesulfonic acid, methyl ester.

The metabolic intermediates of DH5α, E-SDRz, and rSDRz degradation of aniline blue were characterized by Gas chromatography–mass spectrometry (GC-MS), and the results showed that four specific degradation products were detected in the metabolic intermediates of rSDRz degradation of aniline blue. These substances were identified through comparison with substance spectra in databases, confirming them as benzeneacetaldehyde, a, a-diphenyl, 2-Amino-4-methylbenzophenone, benzene, 1-dimethylamino-4-phenylmethyl, benzenesulfonic acid, methyl ester (Figure 8).

The above results preliminarily clarified the SDR’s main metabolic characteristics and pathways involved in aniline blue degradation. The biological degradation of aniline blue by CT1 primarily involves enzymatic catalysis by microorganisms to break the conjugated bond structure of the benzene rings through processes such as oxidation and dehydrogenation. This leads to structural cleavage, breaking the chromophore groups of dye molecules and further degrading complex macromolecular structures into single benzene rings. These were further mineralized into CO_2_ and H_2_O, completing the degradation process.

Currently, the pathways involved in aniline blue degradation by CT1 are not fully understood. However, studies have been reported on the biodegradation of triarylmethane dyes, such as malachite green and aniline blue dyes. For instance, Murugesan et al. identified pathways involving the continuous demethylation and hydroxylation of malachite green catalyzed by laccase (LacA) [42]. Concurrently, Kim et al. isolated a triarylmethane reductase from *Citrobacter* sp. due to its structural similarity to SDR family proteins, and it suggests potential enzymatic similarities in degradation processes [43]. Additionally, Navada’s study on the degradation of aniline blue by endophytic fungi of the *Fusarium* sp. revealed a sequence of oxidation, hydroxylation, deamination, and asymmetric cleavage reactions involved in the process [44]. Shedbalkar et al. proposed a degradation pathway for cotton blue by *Penicillium ochrochloron* MTCC 517, where asymmetric cleavage catalyzed by lignin peroxidase produces sulfonamides and triphenylmethane [45]. These studies indicate that various organisms utilize different enzymes to catalyze the degradation of triarylmethane dyes via analogous enzymatic pathways.

**Figure 9:**
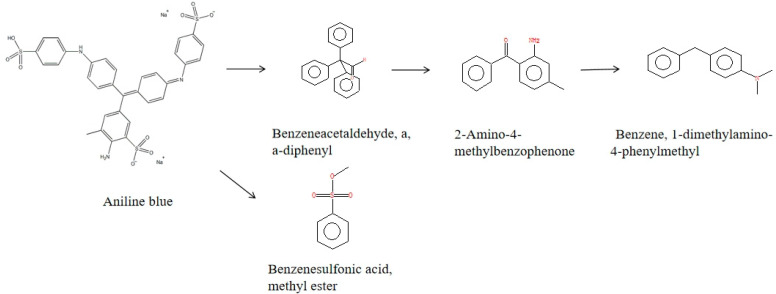
Proposed aniline blue degradation pathway in rSDRz.

Based on biodegradation studies of triarylmethane dyes and analysis of aniline blue degradation products by rSDRz, the primary pathway for SDRz-mediated degradation of aniline blue was inferred (Figure 9). Under the action of rSDRz, both the C=N bond, which is the chromophore group, and the C-N bond of aniline blue are cleaved. At the same time, the sulfonic acid group (-SO_3_H) side chain is also degraded. The central carbon atom of the triarylmethane structure undergoes hydroxylation, leading to the formation of compounds such as benzeneacetaldehyde, a, a-diphenyl, and benzenesulfonic acid, methyl ester. As degradation progresses, the central carbon atom of the triarylmethane structure undergoes further carbonylation, generating benzophenone-like substances such as 2-amino-4-methylbenzophenone, which further cleaves to produce benzene, 1-dimethylamino-4-phenylmethyl.

### 2.9. Environmental Effects and Prospects of Aniline Blue Biodegradation

Aniline blue contaminants in aquatic environments significantly impact ecological systems and agricultural irrigation [46], it is toxic to plants, affecting seed germination, root and seedling growth [8]. Wu et al. assessed plant and genetic toxicity to understand the toxicity of aniline blue and its degradation products on microorganisms and plants [35]. They found that aniline blue dye at a concentration of 800 mg·L^−1^ notably inhibited the stem length of germinating rice seeds. Additionally, at a concentration of 80 mg·L^−1^, aniline blue exhibited potential genotoxicity towards *E. coli* JC19008 [35]. The germination rate of mung bean seeds treated with 3 g·L^−1^ aniline blue was 40 ± 3% [44].

Additionally, the microbial toxicity of aniline blue was assessed on three agricultural microorganisms: *Azospirillum brasilense* (MTCC 4034), *Azotobacter vinelandii* (MTCC 2460), and *Bacillus subtilis* (430). The results revealed significant growth inhibition of all three microorganisms on agar plates, comparable to the bacteriostatic effect observed with the same dose of enrofloxacin (5 μg) [44]. In this study, the effects of aniline blue on mung bean seed germination were investigated. As shown in Figure S11, the plant growth toxicity test results indicated that the aniline blue solution adversely affected both the germination and growth of mung bean seeds. The germination rate of mung beans in the aniline blue solution treatment group was only 68.17%, much lower than 97.56% in the CK group and 92.59% in the aniline blue degradation product group. In the mung bean sprout length measurement after six days of growth, the CK group reached a sprout length of 15.12 cm, while the mung bean sprout length in the aniline blue treatment group (AB2) was only 8.13 cm and in the aniline blue degradation product treatment group (AB1) was 13.28 cm, indicating significant inhibition of mung bean growth by aniline blue, with reduced inhibitory effects after biological degradation of aniline blue on mung bean growth. The results of the toxicity test showed that the degradation products had a reduced toxic effect on the germination and growth of mung bean seeds, which was similar to the findings of Cheng and Wu et al. [8,35], which also indicated that rSDRz played a crucial role in the biodegradation of aniline blue.

The results of our research showed that the oxidoreductase secreted by strain CT1 can destroy the triaryl framework and aromatic ring structure of aniline blue, and the stress tolerant bacteria in the polluted water will work together to completely degrade the aniline blue degradation products, which will eventually be mineralized into carbon dioxide and water, which is also confirmed by the study on the degradation of dye pollutants by other bacteria [47,48]. Gong et al. reported that SDR of strain CT1 activated hsdA gene expression and promotes the degradation of steroid compounds [49]. Matsunaga et al. reported that dehydrogenase/reductase (SDR family) member 4, 11beta-hydroxysteroid dehydrogenase type 1, L-xylulose reductase, two types of aflatoxin B1 aldehyde reductase, participated in the reduced metabolism of various carbonyl compounds which presented in foods, environmental pollutants, and drugs [50]. SDR also plays an important role in the occurrence of toxicity in the degradation of non-coplanar Polychlorinated biphenyls [51]. Therefore, combining the results of metagenomics, meta-transcriptomics, and metabolomics analyses with genetic engineering techniques to enhance microbial degradation, it may play a crucial role in helping to develop effective dye degradation strategies.

In the real-world systems, biodegradation will be more efficient. The use of biodegradation technology can remediate dye pollution in lakes and rivers, and reduce the impact of pollutants on aquatic products and avoid toxic effects on people through bioconcentration. At the same time, biodegradation purifies irrigation water and avoids loss of agricultural production and harm to food crops. Biodegradation is increasingly recognized for its environmental sustainability and its ability to effectively remove a wide range of dye contaminants [52].

## 3. Materials and Methods

### 3.1. Experimental Materials

In this study, seven natural bacterial strains capable of degrading PAHs were selected for the aniline blue degradation experiments. The strains included *C. testosteroni* CT1 (ATCC11996), *C. testosteroni* KF-1, *Buttiauxella* sp. S19-1, *P. putida* PS, *P. stutzeri* JP1, *Pseudomonas* sp. LY1, and *A. calcoaceticus* LM1.

Seven macrocyclic compound-degrading bacteria were selected to compare their abilities to degrade aniline blue. Strain CT1 was identified in 1951 in Barcelona, Spain [53], which can degrade polycyclic aromatic compounds and has been found to contain a series of critical enzymes [22,54]. KF-1 was isolated in Germany, which also is a strain of *C. testosteroni* [32]. PS was isolated from the active sludge of a wastewater treatment plant in Shanghai, China, and is capable of degrading PAHs and trinitrotoluene (TNT) with a high degree of efficiency [55]. S19-1, an effective steroid-degrading strain, was obtained from the Baltic Sea coast. Strain S19-1 was reported to have the ability to degrade TNT and PAHs effectively [56,57]. LM1 and LY1 were isolated from poultry and livestock, which were obtained from the high concentration of PAHs-contaminated sediments in Xiamen Harbor (Xiamen, China) [58], and JP1 was obtained from the sediments of Shantou Bay [59]. These three bacteria could degrade aromatic compounds such as PAHs and estrogen.

*E. coli* DH5α and BL21(DE3) strains were maintained in our laboratory. Plasmids pET-28a, pUcm-T, and antibiotics (kanamycin and ampicillin) were purchased from Sangon Biotech (Shanghai, China). The restriction endonucleases *EcoR* I, *Hind* III, T4 DNA ligase, Taq polymerase, bovine serum albumin, standard gel recovery kits, plasmid miniprep kits, and Ni-TED protein purification affinity chromatography kits were obtained from Sangon Biotech (Shanghai, China). Ampicillin-resistant media contained 100 μg·mL^−1^ ampicillin, and kanamycin-resistant media contained 50 μg·mL^−1^ kanamycin. DNA primer synthesis and sequencing were performed by Meiji Biotechnology Co., Ltd. (Changchun, China). Aniline blue (BR, water-soluble) and Coomassie brilliant blue G-250 (analytical reagenl) were purchased from Macklin Biochemical Co., Ltd. (Shanghai, China). Carbinol and ethyl acetate, both of chromatographic grade, were purchased from Thermo Fisher (China) Scientific (Shanghai, China).

### 3.2. Experimental Methods

#### 3.2.1. Biological Degradation of Aniline Blue by Different Strains

Standard Luria–Bertani (LB) medium was used to culture strains CT1, KF1, and PS (27 °C), and strains S19-1, JP1, LY1, and LM1 (37 °C). Glycerol stocks of 1 mL of bacteria were added to 100 mL of LB medium and incubated overnight at 180 rpm. The optical density at 600 nm (OD_600nm_) of all bacterial cultures was diluted with LB medium to an OD_600nm_ of 1.0. 1 milliliter of the bacterial culture was added to 100 mL of a sterile aqueous solution of 200 mg·L^−1^ aniline blue and incubated at 37 °C for 12 h at 180 rpm. After incubation, the cultures were centrifuged at 5000× *g* for 10 min, and the supernatant was collected to measure optical density at 585 nm (OD_585nm_). The degradation efficiency of the aniline blue dye was calculated using the following formula (1):

(1) $$\eta =(A_{0}-A_{t})/A_{0}\times 100\%$$

where η is the degradation efficiency of aniline blue, A_0_ is the initial absorbance of aniline blue, and A_t_ is the absorbance of aniline blue at time t.

#### 3.2.2. Optimization of Aniline Blue Biodegradation Conditions

One milliliter of the CT1 bacterial culture (OD_600nm_ = 1.0) was inoculated into 100 mL of LB medium containing aniline blue solution. After a certain incubation period, the cultures were centrifuged at 5000× *g* for 10 min, and the supernatant was collected. The OD_585nm_ was measured using a UV-Vis spectrophotometer (Mona, Shanghai, China) to calculate the degradation efficiency of aniline blue. The effects of degradation time (3, 6, 9, 12, 15, and 18 h), incubation temperature (22, 27, 32, and 37 °C), and dye concentration (100 mg·L^−1^, 200 mg·L^−1^, 500 mg·L^−1^, 1000 mg·L^−1^, and 2000 mg·L^−1^) on the degradation of aniline blue by strain CT1 were investigated.

#### 3.2.3. Prokaryotic Transcriptomic Analysis of Aniline Blue Degradation by CT1

Based on the experimental data of strain CT1 degrading aniline blue, prokaryotic transcriptomic sequencing was conducted on CT1 cultured for 12 h with aniline blue concentrations of 200 mg·L^−1^ (CT1_blue2) and 500 mg·L^−1^ (CT1_blue5), as well as on a blank control group (CK_blue) cultured for 12 h without aniline blue. Total RNA was extracted using Bacteria RNA Extraction Kit (Majorbio, Shanghai, China) and genomic DNA was removed. Ribosomal RNA (rRNA) depletion instead of poly(A) purification is performed by a RiboCop rRNA Depletion Kit for Mixed Bacterial Samples (Lexogen, Vienna, Austria, purchased from Beijing SBS Genetech Co., Ltd., Beijing, China) and then all mRNAs were broken into short (200 nt) fragments by firstly adding a fragmentation buffer. Secondly, double-stranded cDNA was synthesized with random hexamer primers (Illumina). When the second strand cDNA was synthesized, dUTP was incorporated in place of dTTP. Then, the synthesized cDNA was subjected to end-repair, phosphorylation, and ‘A’ base addition according to Illumina’s library construction protocol. RNA-seq transcriptome library was prepared following Illumina^®^ Stranded mRNA Prep, Ligation (San Diego, CA) using of total RNA paired-end RNA-seq library was sequenced with the Illumina Novaseq 6000 (Illumina Inc., San Diego, CA, USA). The data generated from Illumina platform were used for bioinformatics analysis. All of the analyses were performed using the free online platform of Majorbio Cloud Platform (www.majorbio.com, accessed on 31 August 2021) from Shanghai Majorbio Bio-pharm Technology Co.,Ltd. Sequencing was performed by Meiji Biotechnology Company. Differential analysis was conducted for CK_blue vs. CT1_blue2, CK_blue vs. CT1_blue5, and CT1_blue2 vs. CT1_blue5. Differential Expression Analysis (DEG) were identified using DESeq 2 software (Version 1.42.0) with the criteria of log_2_FC > 1 (fold change) and significance *p* < 0.05. DEGs were annotated by comparing their sequences with those in the Gene Ontology (GO) and Kyoto Encyclopedia of Genes and Genomes (KEGG) databases.

#### 3.2.4. Construction of SDRz-Expressing Strains

The *SDRz* sequences were obtained from GenBank. Primers pS-F/S-R were designed using Primer 5 software (primer sequences are listed in Table S2). *SDRz* was amplified from strain CT1 using PCR (94 °C for 3 min, 94 °C for 30 s, 50 °C for 45 s, 72 °C for 60 s, for 30 cycles, and 72 °C for 10 min). The amplified gene was ligated into the pUCm-T vector and transformed into *E. coli* DH5α-competent cells. Transformants were selected on an antibiotic-containing solid medium and incubated at 37 °C for 12–16 h. Single colonies were selected for PCR amplification of the *SDRz* gene, and the products were verified by agarose gel electrophoresis. Plasmids from the verified colonies were sequenced, and successful *SDRz*-expressing strains (E-SDRz) were stored at ultra-low temperatures.

#### 3.2.5. Construction of SDRz Knockout Strains

Primers pΔS-F/ΔS-R (Table S2) were designed to amplify the *SDRz* knockout gene (Δ*SDRz*) from the CT1 strain by PCR. The knockout gene was ligated into the pCR2.1-TOPO vector and transformed into *E. coli* DH5α-competent cells. The construct was verified by sequencing the positive clones. The verified plasmid was electroporated into the CT1 strain, and resistant colonies were selected. The *SDRz*-knockout recombinant strain (named CT-ΔSDRz) was identified by PCR (primers pΔS-F’/ΔS-R’, Table S2) verification and sequencing, and the CT-ΔSDRz strain was stored at low temperatures.

#### 3.2.6. Construction of the SDRz Protein Expression Vector

The restriction enzymes *EcoR* I and *Hind* III were used to digest the cloning vector pUCm-T-SDRz (pT-SDRz) and expression vector pET-28a. Target and vector fragments were gel-purified using a standard gel recovery kit. T4 DNA ligase was used to ligate purified products. The ligated product was transformed into *E. coli* BL21(DE3) cells by heat shock and cultured at 37 °C for 16 h. Single colonies were selected and cultured overnight in a LB medium containing kanamycin at 37 °C. Plasmids were extracted, verified using gel electrophoresis, and sequenced. The successful construction of the pET-28a-SDRz protein expression vector (pET-SDRz) was confirmed.

#### 3.2.7. Induction and Purification of rSDRz

The pET-SDRz protein-expressing strain was inoculated into an LB medium containing 50 μg·mL^−1^ kanamycin and cultured overnight at 37 °C. The culture was inoculated into 100 mL of LB medium at a 1:100 ratio and incubated at 37 °C until the OD_600nm_ reached approximately 0.5. The culture was then cooled to 16 °C and induced with IPTG (0.5 mM) for 24 h at the same temperature. The culture was centrifuged at 8000× *g* for 5 min to collect cells. The cells were washed once with PBS and lysed by ultrasonication on ice. The lysate was centrifuged at 14,000× *g* for 30 min at 4 °C, and the supernatant containing the protein was collected. The supernatant was added to a nickel column pre-equilibrated with an equilibration buffer and incubated for 30 min. The column was washed with a wash buffer to remove non-specific proteins and then eluted with elution buffer to obtain the target protein. The purified rSDRz protein was analyzed using 12% SDS-PAGE, and the protein concentration was determined using the Coomassie Brilliant Blue assay.

#### 3.2.8. Functional Verification of SDRz

CT-ΔSDRz and wild-type CT1 cells were activated by overnight culturing. The cultures were inoculated into 10 mL of LB medium at a 1:100 dilution and incubated until the OD_600nm_ reached 1.0. Aniline blue solution was added at a final concentration of 200 mg·L^−1^, and the cultures were incubated at 28 °C with shaking for 12 and 24 h. The cultures were centrifuged at 6000× *g* for 10 min, and the supernatant was collected for OD_578nm_ measurement. The degradation efficiency was calculated using the formula outlined in Section 3.2.1, with the 0 h sample serving as the control. The effect of *SDRz* knockout on aniline blue degradation was analyzed, and degradation experiments were also conducted using *E. coli* DH5α and E-SDRz to assess the impact of SDRz expression on aniline blue degradation.

#### 3.2.9. Enzymatic Properties of rSDRz

To determine the enzymatic activity of rSDRz, the following reaction system was established: 20 mM PB, 500 mM NaCl buffer (pH 7.4), 1 mM NAD(P), 2 mM aniline blue, and 100 μL rSDRz solution (1 mg·mL^−1^). The control reaction mixture did not include an SDRz protein solution. Reactions were performed at 25 °C for 5 min, with substrate conversion indicated by NAD(P)H production, which was measured at 340 nm using a UV-Vis spectrophotometer (Mona, Shanghai, China). One unit of enzymatic activity was defined as the amount of enzymatic activity required to catalyze the conversion of 0.1 mmol NAD(P) per minute under these conditions, and the enzymatic activity was calculated as follows (2):

(2) $$\mathrm{Enzyme}\;\mathrm{activity}=(A_{1}-A_{0})/ℇ\times L\times T$$

A_0_: absorbance value of the control sample; A_1_: absorbance value of the test sample; ℇ: Absorption coefficient of NADH at 340 nm, 6.2 × 10 L·mol^−1^·cm^−1^; L: Measure the optical path of the sample, L = 0.5 cm; T: Reaction time (min).

The kinetic parameters of rSDRz were determined by measuring the enzyme activity of rSDRz. The concentration range was 0.0625~16 mM. GraphPad Prism 8.0.2 (GraphPad Software, Inc., Boston, MA, USA) was used to establish the Mian equation model of a non-sexual regression curve. The experiment was conducted in triplicate.

To evaluate the efficiency of rSDRz in degrading aniline blue, we established a degradation reaction system under the following conditions: 5 mL total volume, pH 7.4, containing 200 mg·L^−1^ aniline blue, 500 μL of rSDRz solution (1 mg·mL^−1^), 20 mM phosphate buffer, and 500 mM NaCl. The reaction mixture was incubated at 28 °C for 5 min, and the UV-Vis absorbance was measured at 578 nm after the reaction. The degradation efficiency was calculated using the following formula, with samples without the SDRz protein solution as controls (3):

(3) $$\mathrm{Degradation}\;\mathrm{efficiency}(\%)=(1-B_{1}-B_{2})\times 100\%$$

where B_1_ is the UV-Vis absorbance of the control sample, and B_2_ is the UV-Vis absorbance of the aniline blue degradation samples.

#### 3.2.10. Detection of Aniline Blue Degradation Products by GC-MS

The products of aniline blue degradation by rSDRz were analyzed using an Agilent 7890 Gas Chromatograph (GC) (Agilent, Santa Clara, CA, USA) coupled with an Agilent 5977A Mass Selective Detector (MSD) (Agilent, USA). The conditions were as follows: separation was performed using an Agilent DB-5 MS column (30 m × 0.25 mm, 0.25 µm) (Agilent, USA). The injection was carried out in splitless mode with a 2 µL volume. The temperature program was 50 °C for 5 min, ramped to 250 °C at 10 °C·min^−1^, and held at 250 °C for 10 min. The injector temperature was maintained at 250 °C, and high-purity helium was used as the carrier gas at a 1 mL·min^−1^ flow rate.

The reaction system (1 mL, pH 7.4) consisted of 200 mg·L^−1^ aniline blue, 0.1 mM NADP, 100 μL rSDRz protein solution (1 mg·mL^−1^), 20 mM PB, and 500 mM NaCl buffer. The reaction mixture was then incubated at 28 °C for 5 min. After the reaction, 1/3 volume of ethyl acetate was added to extract degradation products. Ethyl acetate was evaporated under vacuum, and the degradation products were dissolved in methanol. The solution was filtered through a 0.22 μm organic filter and analyzed by GC-MS to determine the efficiency and products of rSDRz-mediated aniline blue degradation in vitro. Each experiment was conducted in triplicate with three biological replicates.

#### 3.2.11. Toxicity Analysis of Aniline Blue Degradation Products

Seed germination tests were conducted to evaluate the toxicity of aniline blue before and after degradation. 150 mung bean seeds were selected and disinfected with a 2% sodium hypochlorite solution for 1 min, then rinsed five times with distilled water. Fifty seeds were placed in three separate sterile Petri dishes. The first group was treated with sterile water (CK), the second with the degradation solution (rSDRz was used to degrade 200 mg·L^−1^ aniline blue solution for 5 min) of aniline blue (AB1), and the third with 200 mg·L^−1^ aniline blue solution (AB2). The solution was maintained at half the height of the seeds. Seeds treated with sterile water served as controls. Each treatment was conducted in triplicate. The dishes were incubated at 28 °C in a constant-temperature incubator with 85% humidity for six days. The germination rate and shoot length were recorded. The following formula was used to calculate the germination rate (4):

(4) Germination rate (%)=α/β×100%

where α is the number of germinated seeds, and β is the number of seeds tested.

## 4. Conclusions

This study demonstrates the high efficacy of *C. testosteroni* (CT1) in degrading aniline blue, making it a strong candidate for bioremediation of dye-contaminated wastewater. Prokaryotic transcriptomic analysis revealed a significant upregulation of the *SDRz* gene, highlighting its crucial role in aniline blue degradation. Knockout strain CT1-ΔSDRz showed a 1.27-fold reduction in degradation efficiency, while the expression strain E-SDRz exhibited a 1.24-fold increase, confirming the importance of SDRz. GC-MS analysis identified four novel degradation products, suggesting that SDRz initiates degradation by cleaving C=N and C-N bonds, followed by further transformations, leading to less toxic compounds. In conclusion, CT1 effectively degrades aniline blue and reduces its toxicity, offering significant potential for large-scale wastewater treatment. Further research should focus on its application in real-world systems.

## Data Availability

The data will be made available upon request.

## Supplementary Materials

The following supporting information can be downloaded at: https://www.mdpi.com/article/10.3390/molecules29225405/s1, Figure S1: Effect of different conditions on the degradation of aniline blue by strain CT1. A-Effect of degradation time on degradation efficiency of aniline blue. B-Effect of degradation temperature on degradation efficiency of aniline blue. C-Effect of initial concentration on degradation efficiency of aniline blue; Figure S2: Multi-sequence alignment of SDR proteins. A-Best matched proteins from the five reference species: *Comamonas testosteroni*, *Streptomyces, Soybean*, *Bacillales*, *Clostridioides difficile*, *Pseudomonas*; B- Sequence analysis of six similar proteins: WP_003078050.1: SDR family NAD(P)-dependent oxidoreductase, SDRz (*Comamonas testosteroni*), WP 003113501.0: 3-hydroxybutyrate dehydrogenase (*Pseudomonas*), WP 003244042.0: 3-hydroxybutyrate dehydrogenase (*Bacillales*), WP 003538564.1: ABC transporter permease (*Rhizobium*), WP 009890906.1: 3-hydroxybutyrate dehydrogenase (*Clostridioides difficile*), WP 003973892.1 WP 003973892.1: 3-oxoacyl-ACP reductase (*Streptomyces*); Figure S3: Prediction of tertiary structure of *SDRz*; Figure S4: Cloning of the full sequence of *SDRz* genes. A-gel showing size marker (1), PCR of *SDRz* (771 bp, 2); B-gel showing size marker (1), pUCm-T (2773bp, 2) and pUCm-T-SDRz (3544 bp, 2); Figure S5: Blast of sequences between the full sequence of *SDRz* and pUCm-T-SDRz; Figure S6: Cloning of the sequence of Δ*SDRz* genes. A-recombinant identification sequence. Insert bases are labeled, underlined, and displayed in bold; B-gel showing marker (1), PCR of Δ*SDRz* (401 bp, 2); C-Blast of sequences in recombinant. D-gel showing marker (1), PCR of CT-ΔSDRz (240 bp, 2); Figure S7: Construction of *SDRz* expressing-vector with pET28a and pUCm-T-SDRz. A-gel showing size marker (M), double digested product of pET28a (1) and pUCm-T-SDRz(2) by *EcoR* I and *Hind* III. The size of target fragment in pET-28a is approximately 5369 bp, and target gene in pUCm-T-SDRz is approximately 771 bp. B-Blast of sequences between the full sequence of *SDRz* and pET-28a-SDRz; Figure S8: SDS-PAGE analysis of recombinant SDRz. 1: Maker; 2: pET-28a; 3: pET-28a after induced; 4: pET-28a-SDRz after induced; 5: purified rSDRz protein; Figure S9: Standard curve of bovine serum protein; Figure S10: Michaelis-Menten kinetics of rSDRz; Figure S11: Effect of aniline blue and its degradation products on plant growth. A-Germination rate. B-Bud length; Table S1: Statistical table of differential expression of *SDRz* gene in different treatment groups used in this study; Table S2: Primers.
