# Supplementary material for: The Mechanism of Aniline Blue Degradation by Short-Chain Dehydrogenase (SDRz) in Comamonas testosteroni
Source: Molecules. 2024 Nov 15;29(22):5405. doi: 10.3390/molecules29225405 (PMC11597791; doi:10.3390/molecules29225405)
Supplement: Supplementary file 1 [file molecules-29-05405-s001.zip › molecules-3260046-supplementary.pdf]

Supplementary Materials

The mechanism of aniline blue degradation by short chain dehydrogenase (*SDRz*) in *Comamonas testosteroni*

Chuanzhi Zhang, Yong Huang, Jiaxin He, Lei He, Jinyuan Zhang, Lijing Yu, Elshan Musazade, Edmund Maser, Guangming Xiong, Miao Xu *, Liquan Guo *

Figures

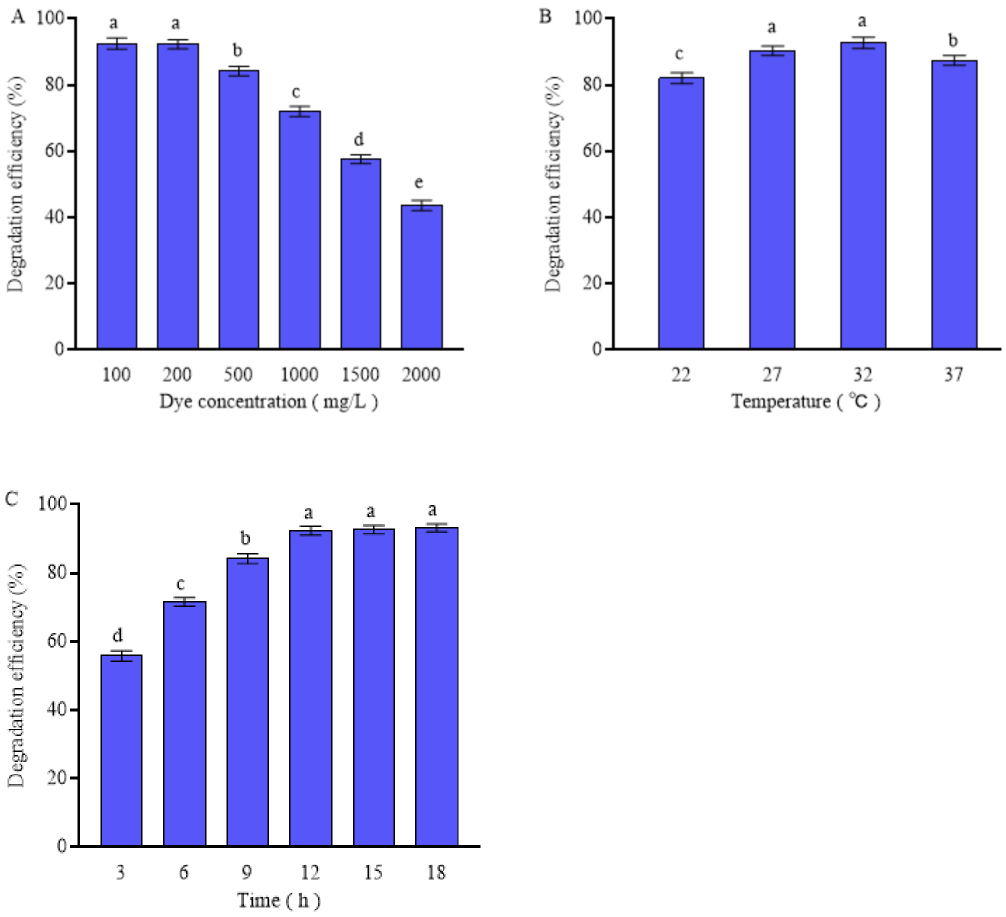

Figure S1 Effect of different conditions on the degradation of aniline blue by strain CT1. A-Effect of degradation time on degradation efficiency of aniline blue. B-Effect of degradation temperature on degradation efficiency of aniline blue. C-Effect of initial concentration on degradation efficiency of aniline blue

A

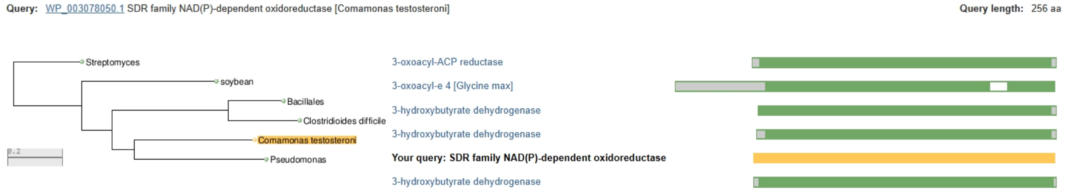

B

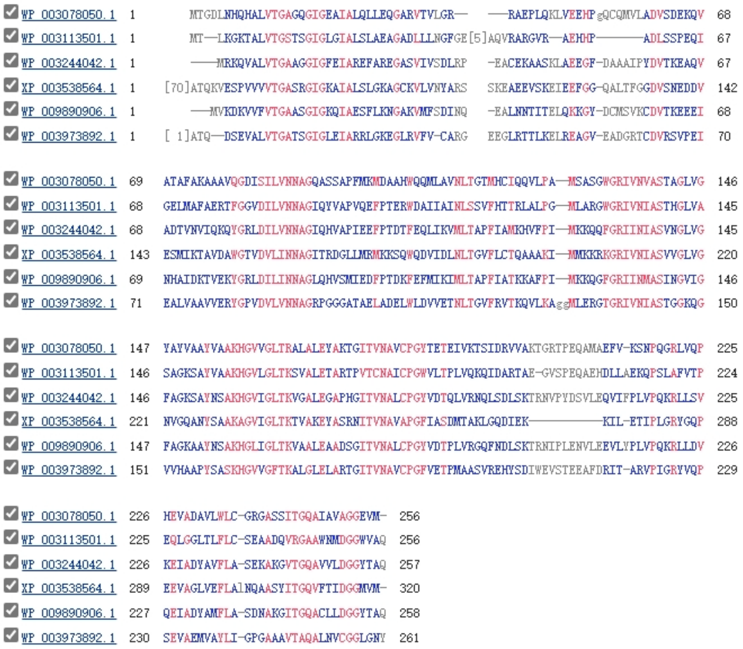

Figure S2 Multi-sequence alignment of SDR proteins.

A-Best matched proteins from the five reference species: *Comamonas testosteroni, Streptomyces, Soybean, Bacillales, Clostridioides difficile, Pseudomonas*.; B- Sequence analysis of six similar proteins: WP_003078050.1: SDR family NAD(P)-dependent oxidoreductase, SDRz (*Comamonas testosteroni*), WP 003113501.0: 3-hydroxybutyrate dehydrogenase (*Pseudomonas*), WP 003244042.0: 3-hydroxybutyrate dehydrogenase (*Bacillales*), WP 003538564.1: ABC transporter permease (*Rhizobium*), WP 009890906.1: 3-hydroxybutyrate dehydrogenase (*Clostridioides difficile*), WP 003973892.1 WP 003973892.1: 3-oxoacyl-ACP reductase (*Streptomyces*)

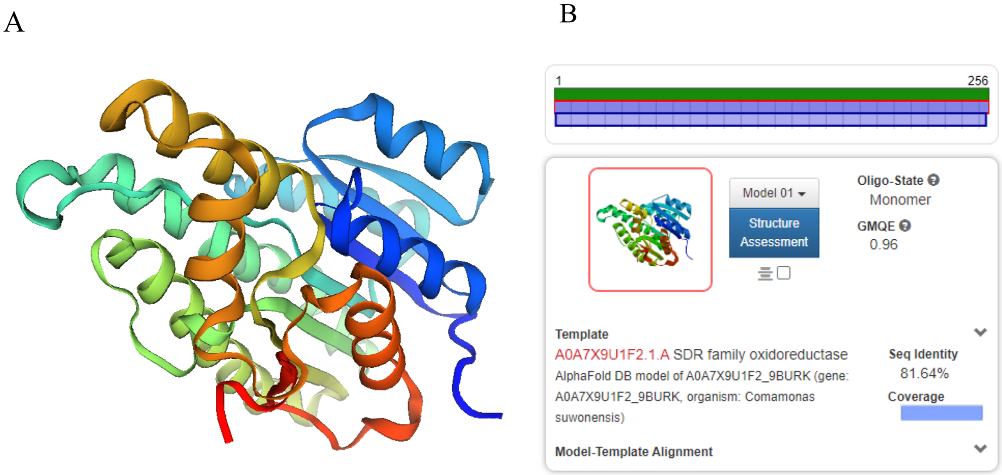

Figure S3 Prediction of tertiary structure of *SDRz*.

A

ATGACAGGCGATCTCAACCATCAACATGCGCTGGTGACGGGCGCGGGCCAGGGCAT
TGGCGAGGCGATTGCGCTCCAACTGCTGGAGCAGGGCGCTCGCGTCACGGTGCTGG
GACGTCGCGCCGAGCCGCTGCAGAAACTGGTGGAAGAGCACCCCGGTCAATGCCAG
ATGGTGCTGGCCGATGTGTCCGATGAAAAACAGGTGGCAACAGCGTTTGCGAAGGC
GGCAGCCGTGCAAGGTGACATCAGCATTCTGGTCAATAACGCCGGCCAGGCCAGCA
GCGCGCCCTTCATGAAGATGGATGCCGCGCATTGGCAGCAGATGCTGGCGGTCAAC
CTGACCGGCACCATGCACTGCATTCAGCAAGTGCTGCCGGCCATGTCCGCTTCGGGC
TGGGGACGCATCGTCAATGTGGCCAGCACGGCTGGTTTGGTGGGCTACGCCTATGTC
GCGGCCTATGTAGCGGCCAAGCATGGCGTGGTGGGCCTGACACGAGCCCTGGCGCT
GGAGTACGCCAAGACCGGGATCACCGTCAACGCCGTCTGCCCCGGCTATACCGAAA
CCGAGATTGTCAAAACCAGCATTGACCGAGTCGTTGCCAAGACCGGCCGCACACCA
GAGCAGGCAATGGCTGAGTTCGTCAAATCCAACCCCCAAGGCCGCCTGGTACAGCC
GCACGAGGTGGCGGACGCCGTGCTGTGGCTATGTGGGCGAGGTGCATCAAGTATTA
CCGGGCAAGCCATAGCCGTCGCTGGTGGCGAAGTCATGTGA

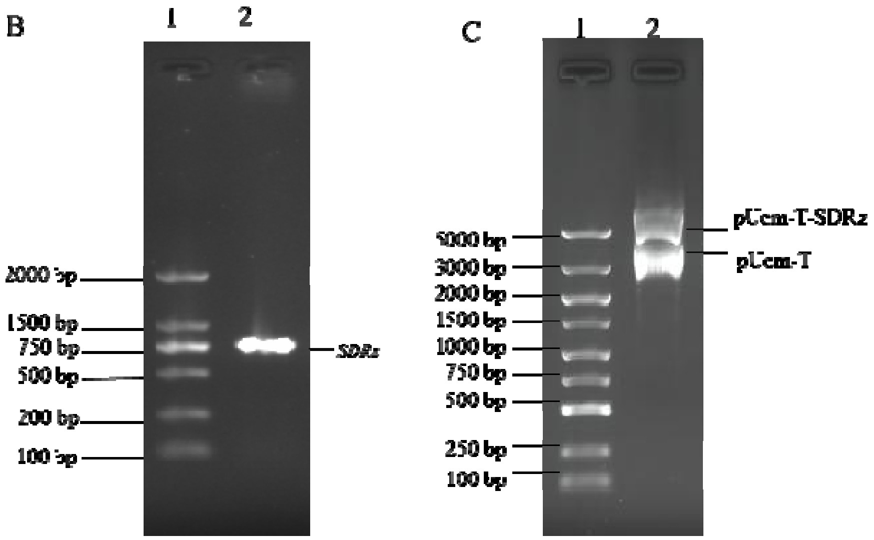

Figure S4 Cloning of the full sequence of *SDRz* genes. A-Sequence of *SDRz*; B-gel showing size marker (1), PCR of *SDRz* (771 bp, 2); C-gel showing size marker (1), pUCm-T (2773bp, 2) and pUCm-T-SDRz (3544 bp, 2).

```
Score              Expect  Identities      Gaps        Strand
1419 bits(768)     0.0     768/768(100%)   0/768(0%)   Plus/Minus

Query  1    ATGACAGGCGATCTCAACCATCAACATGCGCTGGTGACGGGCGCGGGCCAGGGCATTGGC  60
            ||||||||||||||||||||||||||||||||||||||||||||||||||||||||||||
Sbjct  858  ATGACAGGCGATCTCAACCATCAACATGCGCTGGTGACGGGCGCGGGCCAGGGCATTGGC  799

Query  61   GAGGCGATTGCGCTCCAACTGCTGGAGCAGGGCGCTCGCGTCACGGTGCTGGGACGTCGC  120
            ||||||||||||||||||||||||||||||||||||||||||||||||||||||||||||
Sbjct  798  GAGGCGATTGCGCTCCAACTGCTGGAGCAGGGCGCTCGCGTCACGGTGCTGGGACGTCGC  739

Query  121  GCCGAGCCGCTGCAGAAACTGGTGGAAGAGCACCCCGGTCAATGCCAGATGGTGCTGGCC  180
            ||||||||||||||||||||||||||||||||||||||||||||||||||||||||||||
Sbjct  738  GCCGAGCCGCTGCAGAAACTGGTGGAAGAGCACCCCGGTCAATGCCAGATGGTGCTGGCC  679

Query  181  GATGTGTCCGATGAAAAACAGGTGGCAACAGCGTTTGCGAAGGCGGCAGCCGTGCAAGGT  240
            ||||||||||||||||||||||||||||||||||||||||||||||||||||||||||||
Sbjct  678  GATGTGTCCGATGAAAAACAGGTGGCAACAGCGTTTGCGAAGGCGGCAGCCGTGCAAGGT  619

Query  241  GACATCAGCATTCTGGTCAATAACGCCGGCCAGGCCAGCAGCGCGCCCTTCATGAAGATG  300
            ||||||||||||||||||||||||||||||||||||||||||||||||||||||||||||
Sbjct  618  GACATCAGCATTCTGGTCAATAACGCCGGCCAGGCCAGCAGCGCGCCCTTCATGAAGATG  559

Query  301  GATGCCGCGCATTGGCAGCAGATGCTGGCGGTCAACCTGACCGGCACCATGCACTGCATT  360
            ||||||||||||||||||||||||||||||||||||||||||||||||||||||||||||
Sbjct  558  GATGCCGCGCATTGGCAGCAGATGCTGGCGGTCAACCTGACCGGCACCATGCACTGCATT  499

Query  361  CAGCAAGTGCTGCCGGCCATGTCCGCTTCGGGCTGGGGACGCATCGTCAATGTGGCCAGC  420
            ||||||||||||||||||||||||||||||||||||||||||||||||||||||||||||
Sbjct  498  CAGCAAGTGCTGCCGGCCATGTCCGCTTCGGGCTGGGGACGCATCGTCAATGTGGCCAGC  439

Query  421  ACGGCTGGTTTGGTGGGCTACGCCTATGTCGCGGCCTATGTAGCGGCCAAGCATGGCGTG  480
            ||||||||||||||||||||||||||||||||||||||||||||||||||||||||||||
Sbjct  438  ACGGCTGGTTTGGTGGGCTACGCCTATGTCGCGGCCTATGTAGCGGCCAAGCATGGCGTG  379

Query  481  GTGGGCCTGACACGAGCCCTGGCGCTGGAGTACGCCAAGACCGGGATCACCGTCAACGCC  540
            ||||||||||||||||||||||||||||||||||||||||||||||||||||||||||||
Sbjct  378  GTGGGCCTGACACGAGCCCTGGCGCTGGAGTACGCCAAGACCGGGATCACCGTCAACGCC  319

Query  541  GTCTGCCCCGGCTATACCGAAACCGAGATTGTCAAAACCAGCATTGACCGAGTCGTTGCC  600
            ||||||||||||||||||||||||||||||||||||||||||||||||||||||||||||
Sbjct  318  GTCTGCCCCGGCTATACCGAAACCGAGATTGTCAAAACCAGCATTGACCGAGTCGTTGCC  259

Query  601  AAGACCGGCCGCACACCAGAGCAGGCAATGGCTGAGTTCGTCAAATCCAACCCCCAAGGC  660
            ||||||||||||||||||||||||||||||||||||||||||||||||||||||||||||
Sbjct  258  AAGACCGGCCGCACACCAGAGCAGGCAATGGCTGAGTTCGTCAAATCCAACCCCCAAGGC  199

Query  661  CGCCTGGTACAGCCGCACGAGGTGGCGGACGCCGTGCTGTGGCTATGTGGGCGAGGTGCA  720
            ||||||||||||||||||||||||||||||||||||||||||||||||||||||||||||
Sbjct  198  CGCCTGGTACAGCCGCACGAGGTGGCGGACGCCGTGCTGTGGCTATGTGGGCGAGGTGCA  139

Query  721  TCAAGTATTACCGGGCAAGCCATAGCCGTCGCTGGTGGCGAAGTCATG  768
            ||||||||||||||||||||||||||||||||||||||||||||||||
Sbjct  138  TCAAGTATTACCGGGCAAGCCATAGCCGTCGCTGGTGGCGAAGTCATG  91
```

Figure S5 Blast of sequences between the full sequence of *SDRz* and pUCm-T-SDRz.

A

Recombinant identification sequence (401 bp)

ATG**<u>G</u>**ACAGGCGATCTCAACCATCAAC
ATGCGCTGGTGACGGGCGCGGGCCA
GGGCATTGGCGAGGCGATTGCGCTCC
AACTGCTGGAGCAGGGCGCTCGCGTC
ACGGTGCTGGGACGTCGCGCCGAGCC
GCTGCAGAAACTGGTGGAAGAGCAC
CCCGGTCAATGCCAGATGGTGCTGGC
CGATGTGTCCGATGAAAAACAGGTG
GCAACAGCGTTTGCGAAGGCGGCAG
CCGTGCAAGGTGACATCAGCATTCTG
GTCAATAACGCCGGCCAGGCCAGCA
GCGCGCCCTTCATGAAGATGGATGCC
GCGCATTGGCAGCAGATGCTGGCGGT
CAACCTGACCGGCACCATGCACTGCA
TTCAGCAAGTGCTGCCGGCCATGTCC
GCTTCGGGCTGGGGAC

B

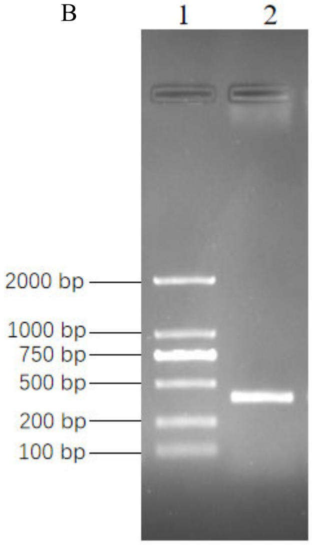

C

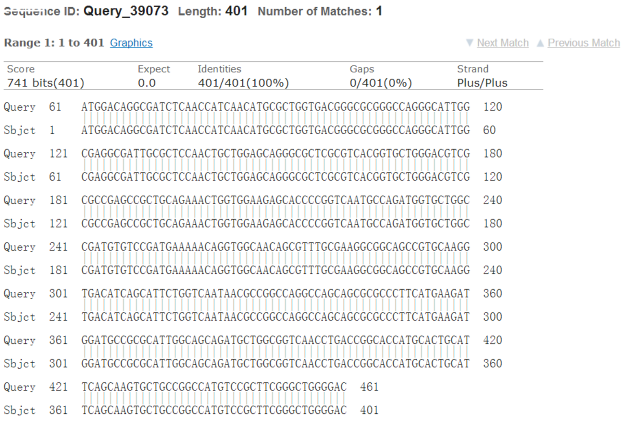

D

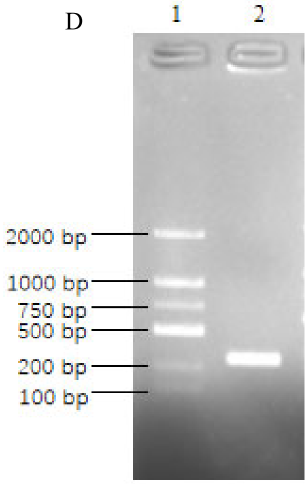

Figure S6 Cloning of the sequence of Δ*SDRz* genes. A-recombinant identification sequence. Insert bases are labelled, underlined, and displayed in bold; B-gel showing marker (1), PCR of Δ*SDRz* (401 bp, 2); C-Blast of sequences in recombinant. D-gel showing marker (1), PCR of CT-ΔSDRz (240 bp, 2).

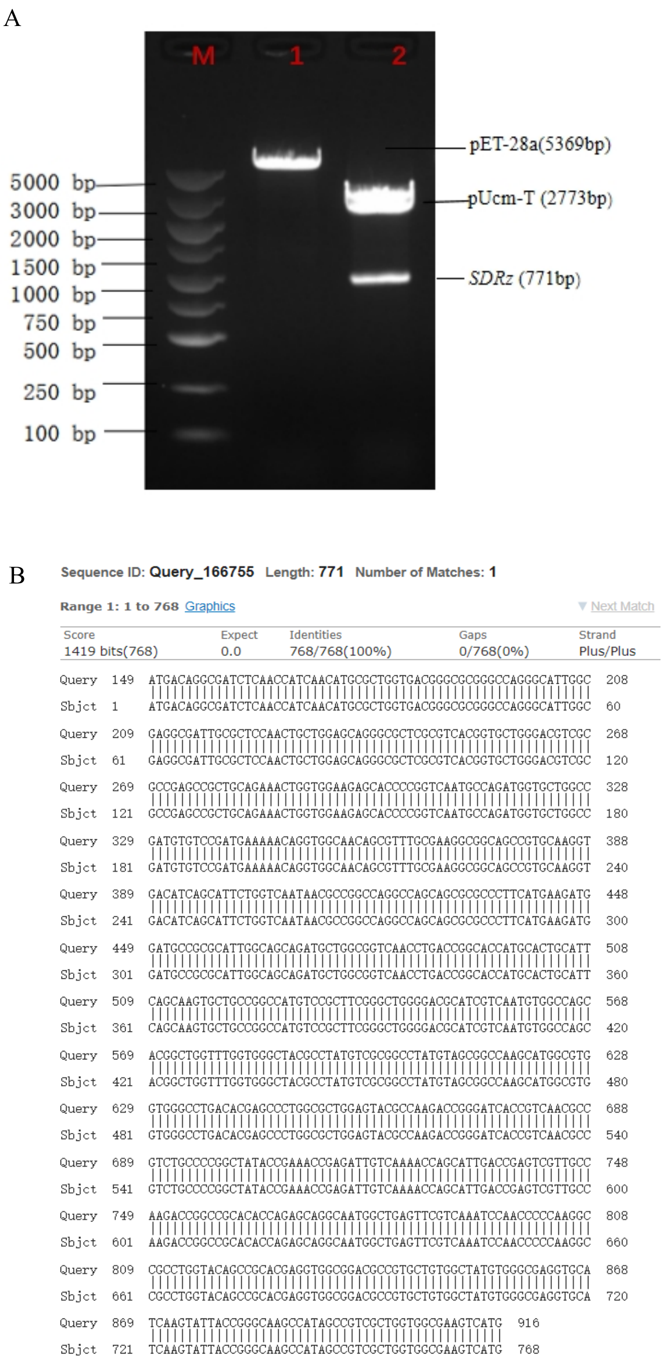

Figure S7 Construction of *SDRz* expressing-vector with pET28a and pUCm-T-SDRz. A-gel showing size marker (M), double digested product of pET28a (1) and pUCm-T-SDRz(2) by *EcoR* Ⅰ and *Hind* Ⅲ. The size of target fragment in pET-28a is approximately 5369 bp, and target gene in pUCm-T-SDRz is approximately 771 bp. B-Blast of sequences between the full sequence of *SDRz* and pET-28a-SDRz.

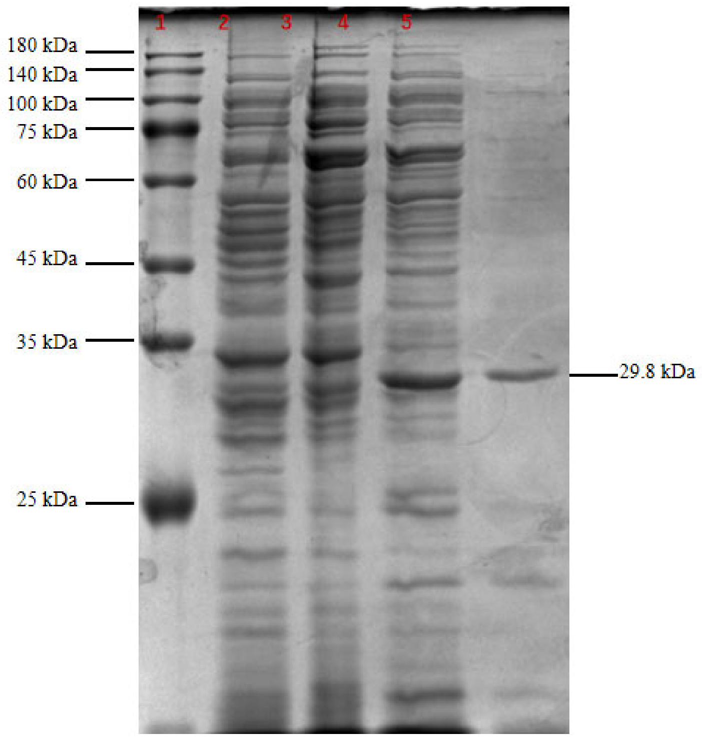

Figure S8 SDS-PAGE analysis of recombinant SDRz .
1: Maker; 2: pET-28a; 3: pET-28a after induced; 4: pET-28a-SDRz after induced; 5: purified rSDRz protein

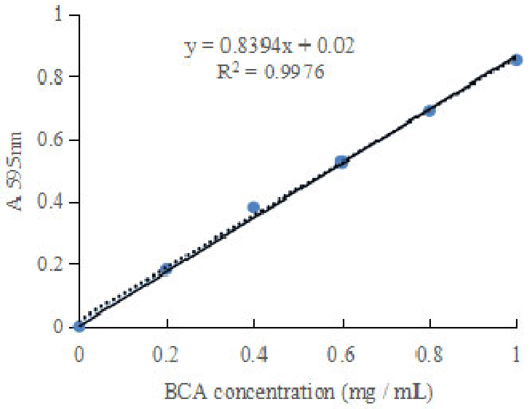

Figure S9 Standard curve of bovine serum protein.

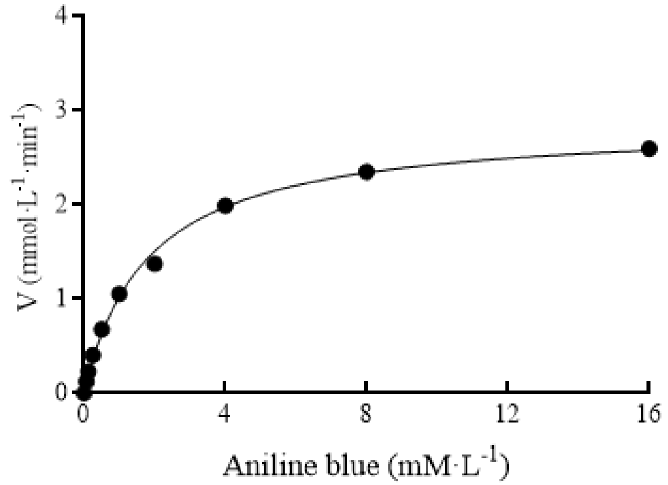

Figure S10 Michaelis-Menten kinetics of rSDRz

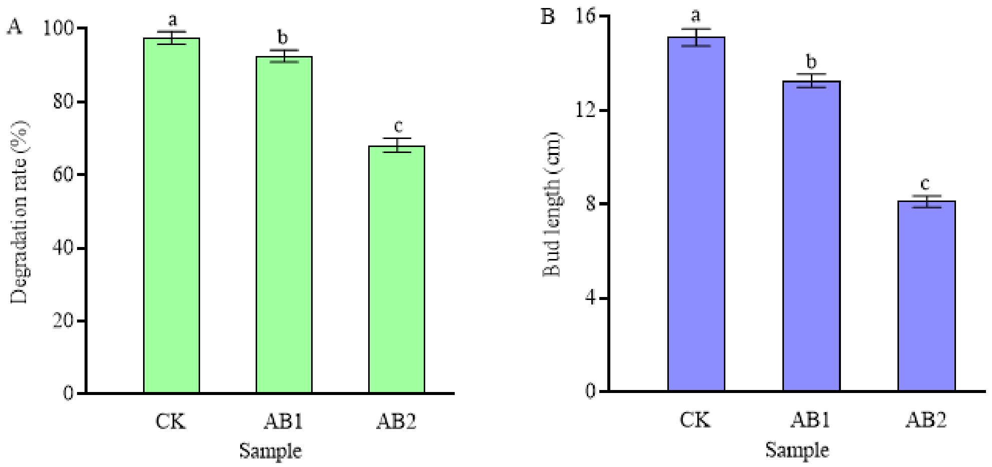

Figure S11. Effect of aniline blue and its degradation products on plant growth. A-Germination rate. B-Bud length.

Tables

Table S1. Statistical table of differential expression of *SDRz* gene in different treatment groups

Sample Name	FC(CT1_blue5/ CK_blue)	Log_2FC(CT1_blue 5/CK_blue)	Regulate	Significant	CK_blue	CT1_blue5
CT1_Blue_5	1.899	0.91233	up	yes	58.69	111.44
CT1_Blue_2	2.156	1.089757	up	yes	58.69	126.52

Table S2 Primers used in this study

Primer name	Sequence
pS-F	CCCATGACAGGCGATCTCAACC
pS-R	CCCCATGACTTCGCCACCAGC
pΔS-F	CCCATGGACAGGCGATCTCAAC
pΔS-R	CCCGTCCCCAGCCCGAAGCGGA
pΔS-F’	CTATCGTCTGGCCCAAGCAATACT
pΔS-R’	TGTTGATGGTTGAGATCGCCTGTC
